# An engineered α-1,3-glucanase-derived probe enables sensitive detection of α-1,3-glucan and reveals its soluble release by *Aspergillus*

**DOI:** 10.1016/j.isci.2026.117952

**Published:** 2026-10-26

**Authors:** Kento Suzuki, Masahiro Kimura, Ken Miyazawa, Yoshiyuki Adachi, Daisuke Yamanaka

**Affiliations:** 1 Laboratory for Immunopharmacology of Microbial Products, School of Pharmacy, Tokyo University of Pharmacy and Life Sciences, Hachioji, Tokyo 192-0392, Japan; 2 School of Bioscience and Biotechnology, Tokyo University of Technology, Hachioji, Tokyo 192-0982, Japan; 3 Department of Fungal Infection, National Institute of Infectious Diseases, Japan Institute for Health Security, Tokyo 162-8640, Japan

**Keywords:** α-1,3-glucan, *Aspergillus*, α-1,3-glucanase, glycoside hydrolase, fungal diagnostics, aspergillosis

## Abstract

α-1,3-Glucan is a major component of the *Aspergillus* cell wall. However, unlike β-1,3-glucan and galactomannan, α-1,3-glucan was not previously considered to be released in a soluble form. Therefore, its potential as a diagnostic biomarker has remained largely unexplored. In this study, we engineered α-1,3-glucanase mutants to develop α-1,3-glucan-specific binding proteins and detection systems. Mutants derived from the endo-type α-1,3-glucanases, AGN1 and AglKA, were generated, among which AglKA-CatD-D1090A lacked enzymatic activity but retained strong binding affinity for α-1,3-glucan. Using this probe, we established a split luciferase assay to detect α-1,3-glucan in *Aspergillus* hyphae and conidia without prior purification. Soluble α-1,3-glucan was substantially released by major *Aspergillus* species, as indicated by results of a sandwich ELISA-like assay with AglKA mutants. These findings suggest that soluble α-1,3-glucan has potential as a complementary diagnostic biomarker for aspergillosis.

## Highlights

• AglKA mutants specifically bind to α-1,3-glucan in the *Aspergillus* cell wall
• A split luciferase assay enables sensitive detection of α-1,3-glucan within 30 min
• An engineered probe detects soluble α-1,3-glucan released by *Aspergillus* species
• Soluble α-1,3-glucan is a potential complementary biomarker for aspergillosis

## Graphical abstract

**Figure:**
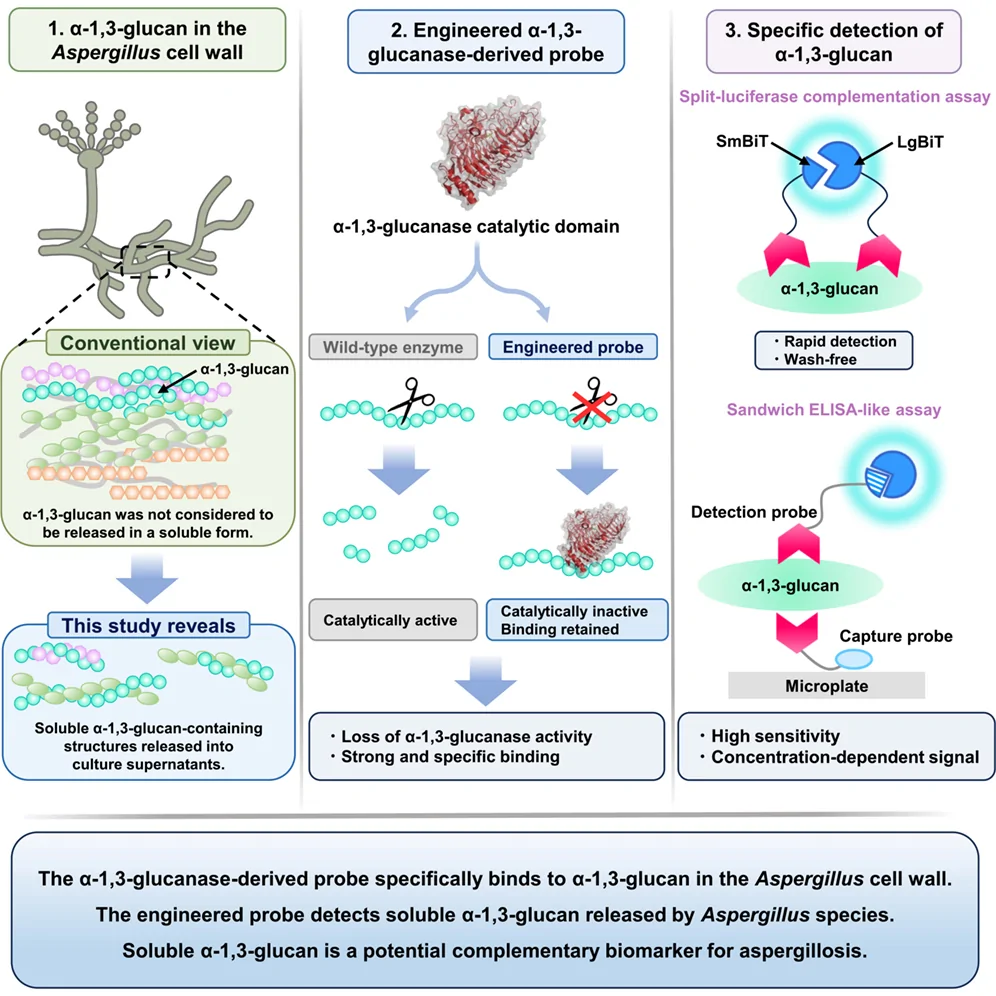

## Teaser

Biochemistry; Bioengineering; Enzyme engineering; Glycobiology; Mycology

## Introduction

Aspergillosis is a major opportunistic fungal infection, along with candidiasis and cryptococcosis.^1^ Exposure to airborne *Aspergillus* spores is common; however, patients with underlying pulmonary diseases or immunocompromising conditions are particularly susceptible to aspergillosis.^2^ The clinical importance of aspergillosis is expected to increase with the increasing aging populations and use of immunosuppressive therapies and invasive medical procedures.^3^^,^^4^ Therefore, sensitive and reliable diagnostic methods are becoming increasingly important.

Over 3 million individuals are affected by chronic pulmonary aspergillosis (CPA), over 4.8 million by allergic bronchopulmonary aspergillosis, and at least 300,000 by invasive aspergillosis (IA).^1^^,^^5^ Mortality rates remain high at approximately 20% for CPA and 90% for IA in critically ill patients.^2^^,^^6^ The clinical manifestations of aspergillosis often overlap with those of mucormycosis, making differential diagnosis challenging.^7^ Early diagnosis and therapy are essential for improving patient outcomes and reducing mortality.^2^^,^^3^ However, currently available diagnostic methods have several limitations.

Serum antigen assays targeting fungal cell-wall polysaccharides are commonly used as adjunct diagnostic tools for the early detection of fungal infections.^8^^,^^9^ For aspergillosis, assays detecting β-1,3-glucan and galactomannan (GM) are routinely used in clinical practice.^10^ However, both assays can be affected by factors that complicate specificity and result interpretation, making their results difficult to interpret in some clinical settings.^11^^,^^12^ Complementary approaches, including *Aspergillus* PCR-based assays and monoclonal-antibody-based tests, such as JF5-based lateral-flow devices and 1D1-based galactosaminogalactan assays, have been developed, reflecting the ongoing need for additional diagnostic tools.^13^^,^^14^^,^^15^^,^^16^ Individual fungal antigen markers differ in sensitivity, specificity, and biological origin; therefore, combined interpretation of multiple polysaccharide antigens may improve diagnostic accuracy.^17^^,^^18^ Additional polysaccharide antigens released by *Aspergillus* may provide complementary information to existing biomarkers and retain the practical advantage of rapid antigen detection. These considerations prompted us to explore *Aspergillus*-derived polysaccharides as potential diagnostic targets.

The outer cell wall of *Aspergillus* species contains considerable α-1,3-glucan, a glucose polymer composed of α-1,3-glycosidic linkages.^19^ Similar α-1,3-glucan structures are also found in the cell walls of several pathogenic fungi, including *Paracoccidioides*, *Histoplasma*, *Blastomyces*, and *Cryptococcus*.^19^ Given its abundance in fungal cell walls and absence in humans, α-1,3-glucan represents a potential target for fungal detection. One consideration in its use as a diagnostic target is that α-1,3-glucan is not exclusive to fungi; for example, mutan, an extracellular polysaccharide produced by cariogenic oral *Streptococcus* species, contains α-1,3-glucan structures.^20^ Therefore, combined detection of multiple fungal cell-wall polysaccharides may improve diagnostic specificity by providing more discriminatory information than single-marker assays. Previously used α-1,3-glucan-binding molecules, such as the mouse monoclonal immunoglobulin M (IgM) antibody MOPC 104E and carbohydrate-binding module (CBM) derived from an α-1,3-glucanase (AglKA-CBM), are valuable for α-1,3-glucan detection.^21^^,^^22^^,^^23^ However, to the best of our knowledge, a highly sensitive α-1,3-glucan detection assay is unavailable. Additionally, the investigation of α-1,3-glucan-related biomarkers has remained largely unexplored. We previously developed high-affinity probes for sensitive detection of β-1,6-glucan and chitin by engineering proteins derived from a fungal endo-β-1,6-glucanase and human chitinases.^24^^,^^25^ Therefore, we investigated whether this strategy could be applied to α-1,3-glucan detection. In this study, we engineered an α-1,3-glucanase-derived probe and established split luciferase complementation and sandwich ELISA platforms for sensitive α-1,3-glucan detection, revealing the release of soluble α-1,3-glucan structures by clinically relevant *Aspergillus* species.

## Results

### Engineering protein probes for α-1,3-glucan recognition

Among the more than 190 glycoside hydrolase (GH) families classified based on amino acid sequence similarity (CAZy database, https://www.cazy.org/), endo-α-1,3-glucanases (EC 3.2.1.59) are restricted to only two families, GH71 and GH87.^26^ Therefore, we selected two representative α-1,3-glucanases, AGN1 from *Schizosaccharomyces pombe* (GH71)^26^ and AglKA from *Bacillus circulans* (GH87),^27^ and generated recombinant variants carrying substitutions at catalytic residues.

For AGN1, the catalytic residues D259 and E262, which are essential for α-1,3-glucan hydrolysis, were substituted with asparagine (N) and glutamine (Q), respectively.^26^ AglKA contains both a catalytic domain (CatD) and a carbohydrate-binding domain. To evaluate whether the catalytic domain could serve as the basis for an α-1,3-glucan detection probe, three CatD variants (D1067A, D1090A, and D1091A) were generated through alanine substitution of residues that are essential for catalysis in AglKA.^27^ To facilitate sensitive detection and minimize interference with the probe function, the recombinant proteins were fused to the small HiBiT peptide tag. HiBiT is a luminescence-based reporter that complements LgBiT, a nonluminescent fragment, to form an active luciferase (NanoLuc).^28^ This approach was successfully used in previous chitin detection assays.^25^ Accordingly, HiBiT-tagged α-1,3-glucanase variants were constructed and expressed in *Escherichia coli* (Figure 1A). For comparison, AglKA-CBM, a previously reported α-1,3-glucan detection probe based on a carbohydrate-binding module,^22^ was also constructed as a HiBiT-tagged fusion protein. Purified recombinant proteins appeared as a single band at the expected molecular weight as indicated with sodium dodecyl sulfate-polyacrylamide gel electrophoresis (SDS-PAGE) (Figures S1A and S1B).

**Figure 1:**
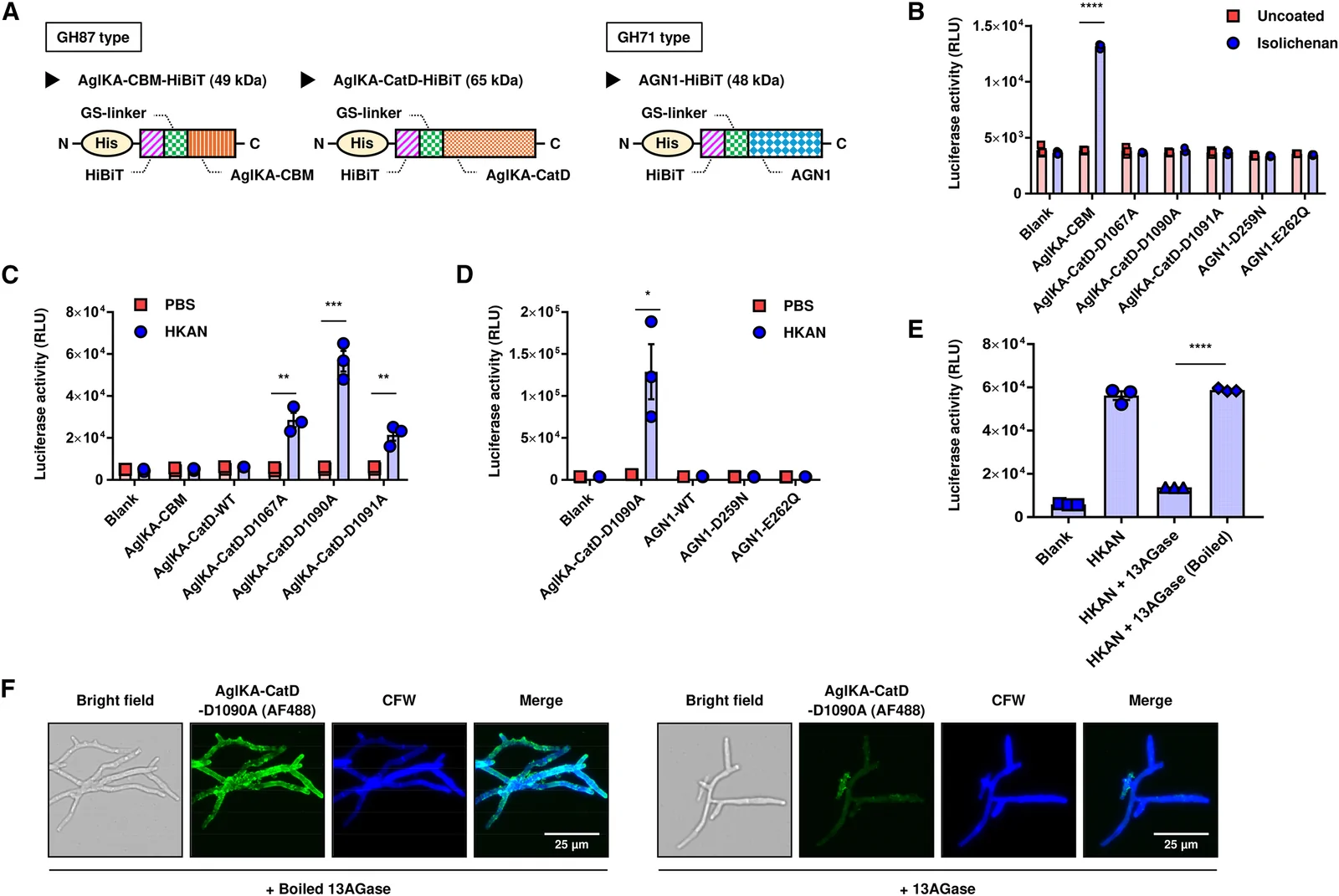
Screening of α-1,3-glucanase-derived probes for binding to *Aspergillus* α-1,3-glucan (A) Structural schematic of α-1,3-glucanase-derived probes. (B) Direct ELISA analysis of α-1,3-glucanase-derived proteins binding to immobilized isolichenan (1 μg/mL). Data are presented as mean ± SEM of three technical replicates (*n* = 3). ∗∗∗∗*p* < 0.0001: significant difference vs. uncoated well (Bonferroni’s multiple-comparisons test). (C and D) Binding of (C) AglKA-derived and (D) AGN1-derived constructs to heat-killed *Aspergillus nidulans* (HKAN) mycelia was assessed using mycelium-associated HiBiT luminescence after removal of unbound protein. Data are presented as mean ± SEM of three independent biological replicates (*n* = 3). ∗*p* < 0.05, ∗∗*p* < 0.01, ∗∗∗*p* < 0.001: significant difference vs. PBS (Bonferroni’s multiple-comparisons test). (E) Effect of HKAN pretreatment with active or heat-inactivated α-1,3-glucanase (13AGase) on AglKA-CatD-D1090A binding. Data are presented as mean ± SEM of three technical replicates (*n* = 3). ∗∗∗∗*p* < 0.0001: significant difference between active and inactivated 13AGase (Welch’s *t* test). (F) Fluorescence microscopy of AglKA-CatD-D1090A-HiBiT binding to HKAN after active or heat-inactivated α-1,3-glucanase (13AGase) treatment. Cell wall chitin was stained with Calcofluor White (CFW). Probe binding was detected using an Alexa Fluor 488 (AF488)-conjugated anti-His-tag antibody. Scale bars, 25 μm. (B–E) Representative data from at least two independent experiments are shown.

Next, α-1,3-glucan was prepared from sucrose using a glucosyltransferase as previously described.^29^ Loss of glycolytic activity was assessed using fluorophore-assisted carbohydrate electrophoresis of the degradation products generated after incubation of the recombinant enzymes (AglKA and AGN1) with α-1,3-glucan.^30^ The wild-type enzymes generated readily detectable oligosaccharides, whereas degradation products by all variants were below the detection limit (Figure S2).

We evaluated the α-1,3-glucan-binding activity of each probe using a direct ELISA-like assay. α-1,3-Glucan is typically insoluble in water; therefore, commercially available isolichenan, a lichen-derived water-soluble α-1,3-/1,4-glucan containing α-1,3-linked regions, was used as the immobilized ligand for ELISA. Among the variants tested, only AglKA-CBM exhibited detectable binding in the initial screening with isolichenan, whereas AglKA-CatD mutants and AGN1 mutants showed little or no binding activity (Figure 1B).

Following the initial screening using a soluble model substrate, we evaluated probe binding to native fungal cell wall α-1,3-glucan using heat-killed *Aspergillus nidulans* (HKAN) mycelium. A binding-dependent detectable increase in HiBiT-derived luminescence was only observed for the AglKA-CatD variants, whereas wild-type AglKA-CatD, AglKA-CBM, and all AGN1-derived constructs remained below the detection limit. Based on its highest luminescence signal among the AglKA-CatD variants (Figures 1C and 1D), AglKA-CatD-D1090A was selected for subsequent characterization. To further assess binding specificity, HKAN was pretreated with active or heat-inactivated α-1,3-glucanase before probing with AglKA-CatD-D1090A. Only the active enzyme significantly reduced binding (Figure 1E).

Probe localization was also examined using similarly treated HKAN samples incubated with AglKA-CatD-D1090A-HiBiT, followed by fluorescence microscopy with a secondary antibody. Fluorescence signals from AglKA-CatD-D1090A were observed along the mycelial surface of control HKAN and overlapped with staining using calcofluor white, a chitin-binding dye, whereas the AglKA-CatD-D1090A signals were markedly reduced after treatment with active α-1,3-glucanase (Figure 1F).

### Characterization of AglKA-CatD-D1090A as a probe for α-1,3-glucan detection

The binding affinities of AglKA-derived constructs were measured using biolayer interferometry (BLI)^31^ with sensor tips immobilized with biotinylated α-glucan, which was enzymatically synthesized from sucrose.^29^ As observed in the HKAN binding assay, AglKA-CatD-D1090A produced the strongest response among the constructs tested (Figure 2A). BLI analysis using serial 2-fold dilutions of AglKA-CatD-D1090A yielded an equilibrium dissociation constant of 2.43 × 10^−8^ M (Figure 2B).

**Figure 2:**
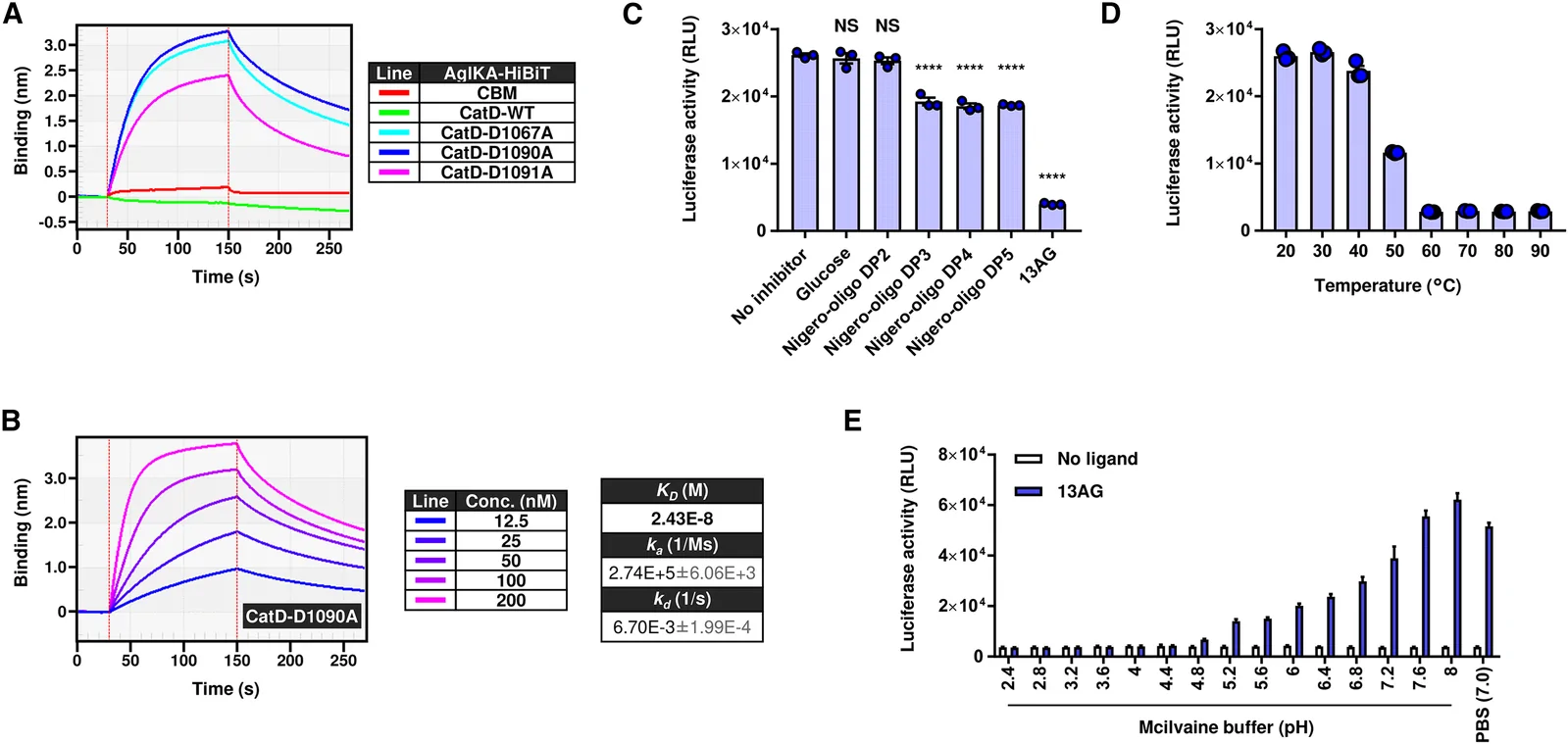
Biochemical characterization of AglKA-CatD-D1090A (A) Binding of AglKA-derived constructs to sensor-immobilized biotinylated α-glucan was analyzed using biolayer interferometry. CatD-WT: wild-type AglKA-CatD. (B) Binding kinetics of AglKA-CatD-D1090A-HiBiT were determined using a 2-fold dilution series (12.5–200 nM), and the equilibrium dissociation constant (*K*_D_) was calculated. (C) The minimal α-1,3-glucan unit recognized by AglKA-CatD-D1090A-HiBiT was evaluated using competitive ELISA with α-glucan-coated plates and glucose, nigero-oligosaccharides (DP2–5), or α-glucan (13AG) as competitors. ∗∗∗∗*p* < 0.0001; NS, not significant: significant difference vs. no inhibitor (Bonferroni’s multiple-comparisons test). (D and E) Thermal and pH stability of AglKA-CatD-D1090A. For thermal stability (D), AglKA-CatD-D1090A-HiBiT was pretreated at 20°C–90°C for 5 min. For pH stability (E), AglKA-CatD-D1090A-HiBiT was incubated in McIlvaine buffer at the indicated pH values. Binding to plate-immobilized α-1,3-glucan (13AG) was measured after each treatment. PBS (pH 7.0) and uncoated wells (no ligand) were included as controls in (E). (C–E) Data are presented as mean ± SEM of three technical replicates (*n* = 3) for (C) and (D) and as mean ± SD of three technical replicates (*n* = 3) for (E). (C–E) Representative data from at least two independent experiments are shown.

Next, to determine the chain-length requirement for AglKA-CatD-D1090A binding, competitive ELISA was performed on α-glucan-coated plates using glucose or α-1,3-linked nigero-oligosaccharides (DP2–5; DP, degree of polymerization) as competitors. The luminescence significantly reduced in the presence of nigero-oligosaccharides of DP3 or higher (Figure 2C). To further characterize AglKA-CatD-D1090A, the thermal and pH stability were assessed using α-glucan-coated plates. Binding was retained after heating up to 40°C, decreased at 50°C, and was lost at 60°C (Figure 2D). It was also retained over a pH range of 4.8–8.0, with maximal binding at pH 8.0 (Figure 2E).

Collectively, these results demonstrate that the alanine substitution at D1090 confers detectable α-1,3-glucan-binding activity on AglKA-CatD. AglKA-CatD-D1090A bound purified α-glucan, recognized α-1,3-linked oligosaccharides of at least three glucose units, and retained binding activity under standard assay conditions, including at room temperature and a broad assay-compatible pH.

### Development of a wash-free homogeneous assay for α-1,3-glucan

To establish a homogeneous assay for α-1,3-glucan detection based on our previously reported split luciferase complementation assay (SLCA) platform,^32^ AglKA-CatD-D1090A was separately fused to SmBiT and LgBiT, the complementary fragments of NanoLuc luciferase (Figure 3A). The resulting two reporter proteins were expressed in *E. coli*, purified, and detected as single bands at their expected molecular weight with SDS-PAGE (Figure 3B; Figure S1C).

**Figure 3:**
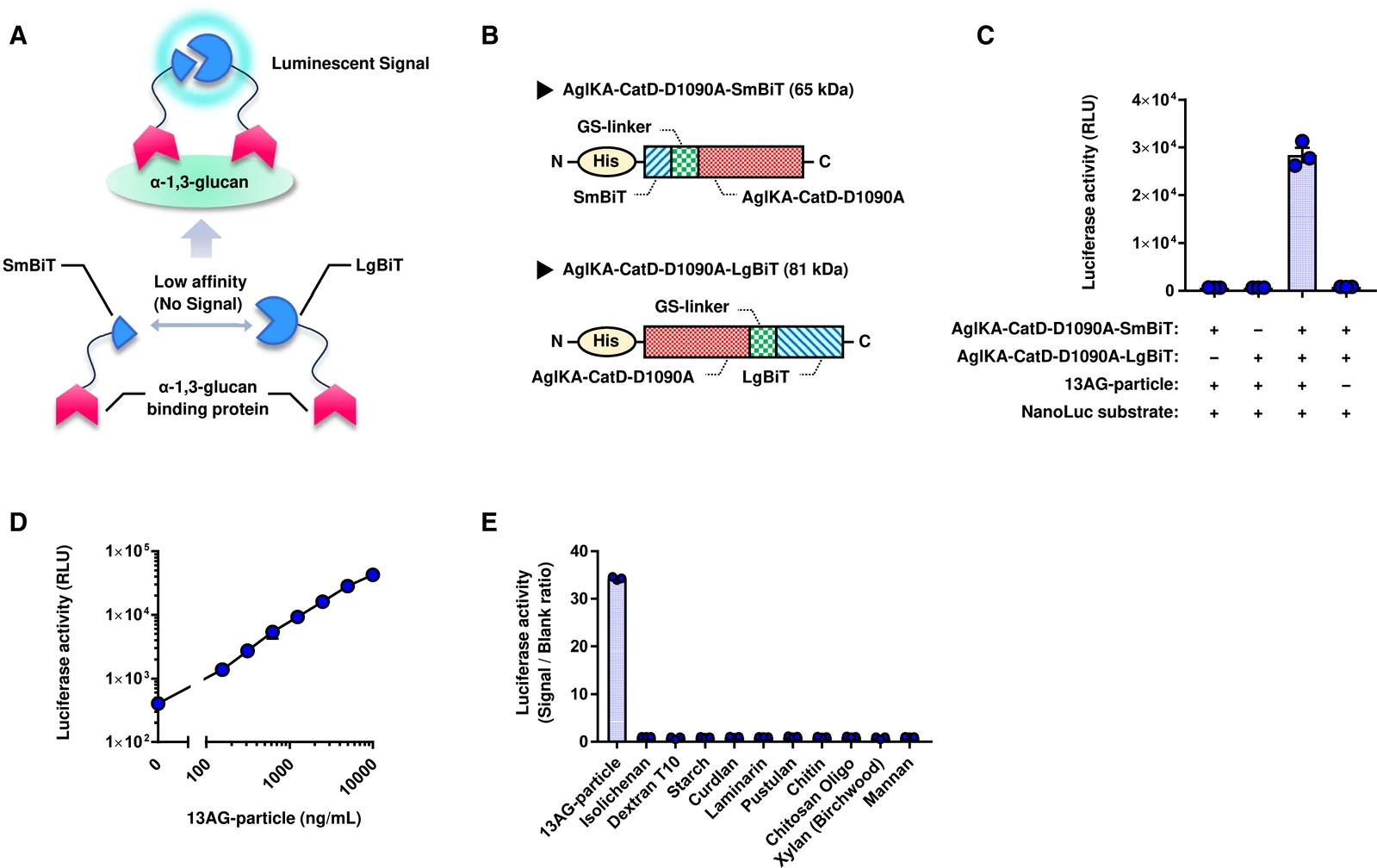
Development and validation of a split luciferase complementation assay for specific α-1,3-glucan detection (A) Schematic of α-1,3-glucan detection using the AglKA-CatD-D1090A-based SLCA. (B) Schematic design of AglKA-CatD-D1090A fused to SmBiT or LgBiT. (C) Requirement of both reporter proteins (200 nM each) and α-1,3-glucan (13AG, 10 μg/mL) for luminescence generation. (D) Dose-dependent luminescence response of the SLCA to synthetic α-1,3-glucan (13AG) particles (0–10,000 ng/mL). (E) Structural specificity of the SLCA against the indicated polysaccharides. Reactivity is expressed as the signal/blank ratio, where blank represents the reaction mixture without a polysaccharide sample. (C–E) Data are presented as mean ± SEM of three technical replicates (*n* = 3) for (C) and (E) and as mean ± SD of three technical replicates (*n* = 3) for (D). (C–E) Representative data from at least two independent experiments are shown.

The two reporter proteins were incubated for 30 min with synthetic α-1,3-glucan particles, and the luminescence was measured after addition of the NanoLuc substrate. The Luminescence was detected only when both reporter proteins and α-1,3-glucan were present (Figure 3C). The signal increased in an α-1,3-glucan concentration-dependent manner (Figure 3D).

To assess the structural specificity of the SLCA, the reactivity across a range of non-target polysaccharides was evaluated. No detectable luminescence was observed with isolichenan (α-1,3-/α-1,4-glucan), starch (α-1,4-glucan), dextran (α-1,6-glucan), curdlan (β-1,3-glucan), laminarin (β-1,3-/β-1,6-glucan), pustulan (β-1,6-glucan), xylan (β-1,4-glucan), mannan, chitin, or chitosan (Figure 3E). Therefore, an AglKA-CatD-D1090A-based, rapid, and wash-free homogeneous SLCA for highly specific detection of α-1,3-glucan was established.

### Validation of the SLCA for α-1,3-glucan detection in *Aspergillus* samples

Next, we evaluated whether the AglKA-CatD-D1090A-based SLCA could directly detect α-1,3-glucan on unpurified fungal cells. We observed dose-dependent luminescence responses to heat-killed mycelia of *A. nidulans*, *Aspergillus fumigatus*, *Aspergillus flavus*, and *Aspergillus terreus* over 0–1,000 μg/mL (Figure 4A). In contrast, no detectable responses were observed with heat-killed *Candida albicans* (yeast form) and *Rhizopus oryzae* (mycelium) (data not shown), in which α-1,3-glucan is absent.^33^^,^^34^

**Figure 4:**
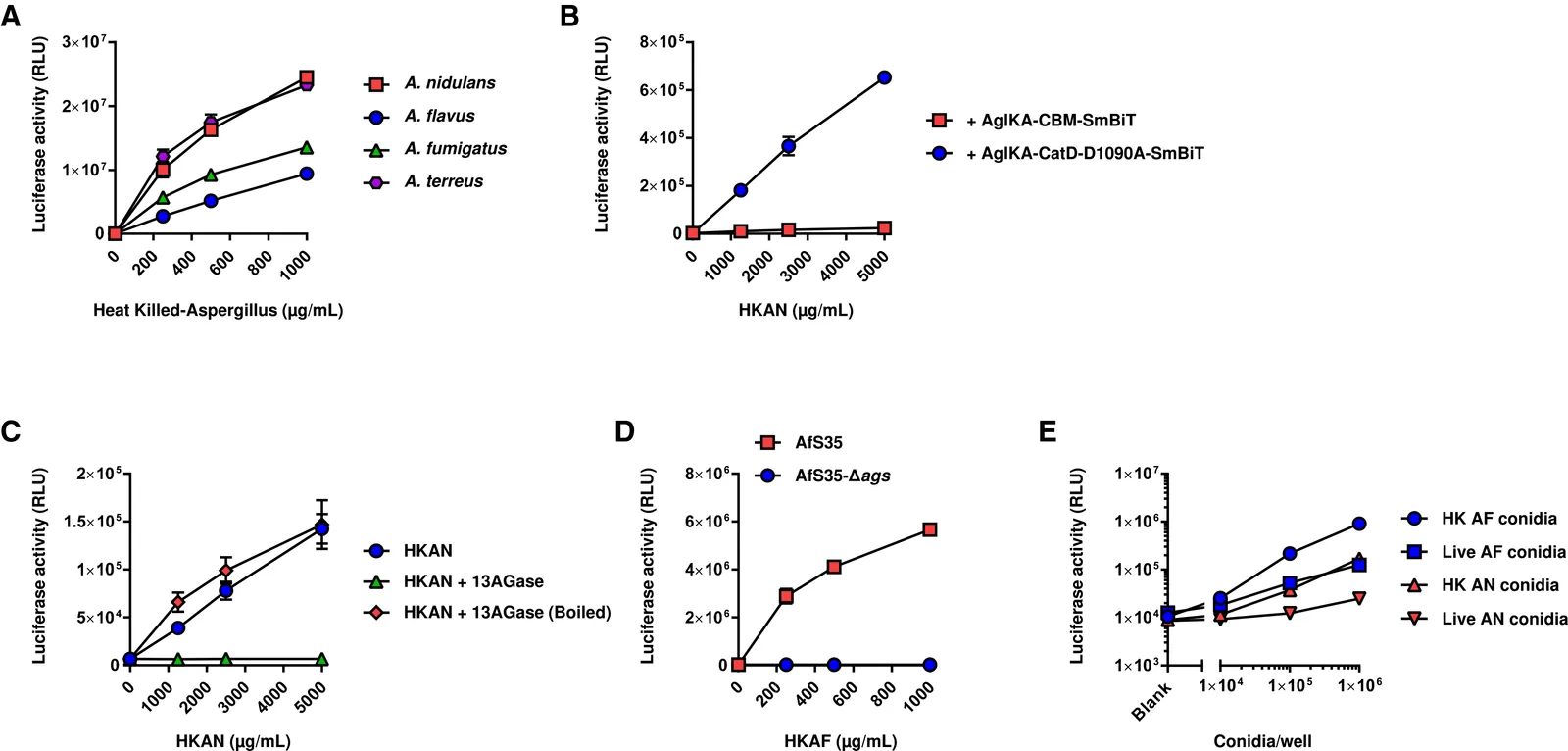
Specific detection of α-1,3-glucan in *Aspergillus mycelia* and conidia by AglKA-CatD-D1090A-based SLCA (A) Dose-dependent SLCA responses to heat-killed mycelia of *A. nidulans*, *A. fumigatus*, *A. flavus*, and *A. terreus* (0–1,000 μg/mL). (B) Comparison of SLCA responses using AglKA-CatD-D1090A-SmBiT or AglKA-CBM-SmBiT paired with AglKA-CatD-D1090A-LgBiT against heat-killed *A. nidulans* mycelia (HKAN). (C) SLCA responses to untreated HKAN and HKAN pretreated with active or heat-inactivated α-1,3-glucanase (13AGase). (D) SLCA responses to heat-killed mycelia of the parental *A. fumigatus* (AfS35) and α-1,3-glucan-deficient (AfS35-Δ*ags*) strain. (E) SLCA responses to untreated and heat-killed conidia of *A. fumigatus* and *A. nidulans* (AF, *A. fumigatus*; AN, *A. nidulans*; HK, heat-killed). (A–E) Data are presented as mean ± SD of three technical replicates (*n* = 3) and are representative of at least two independent experiments. SLCA, split luciferase complementation assay.

For comparison with the conventional α-1,3-glucan detection tool, AglKA-CBM, an AglKA-CBM-SmBiT reporter, was prepared and paired with AglKA-CatD-D1090A-LgBiT. This CBM-containing reporter pair showed lower luminescence signals against HKAN than those of the AglKA-CatD-D1090A-only pair, demonstrating the superior detection capability of AglKA-CatD-D1090A (Figure 4B).

To confirm α-1,3-glucan-specific detection using the SLCA, HKAN was pretreated with active or heat-inactivated α-1,3-glucanase before analysis. We observed dose-dependent luminescence with the untreated and heat-inactivated enzyme-treated HKAN but not with active enzyme-treated HKAN (Figure 4C). Consistently, heat-killed mycelia of an *A. fumigatus* *ags1*/*ags2*/*ags3* triple-knockout strain (AfS35-Δ*ags*; lacking *ags1*, *ags2*, and *ags3*)^35^ was not detected, unlike the parental strain (AfS35) (Figure 4D).

The cell wall polysaccharides of ungerminated conidia are typically masked by melanin and rodlet layers.^33^ We observed dose-dependent responses to untreated conidia of *A. fumigatus* and *A. nidulans*, which were significantly enhanced in heat-killed conidia (Figure 4E). This enhancement may reflect increased exposure of α-1,3-glucan caused by the heat-mediated disruption of the conidial surface layers. These results establish the rapid SLCA as a reliable tool for sensitive and specific detection of cell wall α-1,3-glucan.

### Detection of α-1,3-glucan structures in the soluble fraction of *Aspergillus* culture supernatants using SLCA

Next, we examined whether *Aspergillus* releases α-1,3-glucan structures into culture supernatants. Native α-1,3-glucan is typically insoluble; therefore, supernatants were passed through a 0.45-μm filter before SLCA analysis. The AglKA-CatD-D1090A-based SLCA detected dilution-dependent luminescent signals in *A. nidulans* culture supernatants, whereas only background noise levels were observed for the control medium (Figure 5A). The signals were markedly reduced with the active α-1,3-glucanase pretreatment but not with heat-inactivated α-1,3-glucanase or β-1,3-glucanase, confirming the dependence of the SLCA signals on α-1,3-glucan structures (Figure 5B).

**Figure 5:**
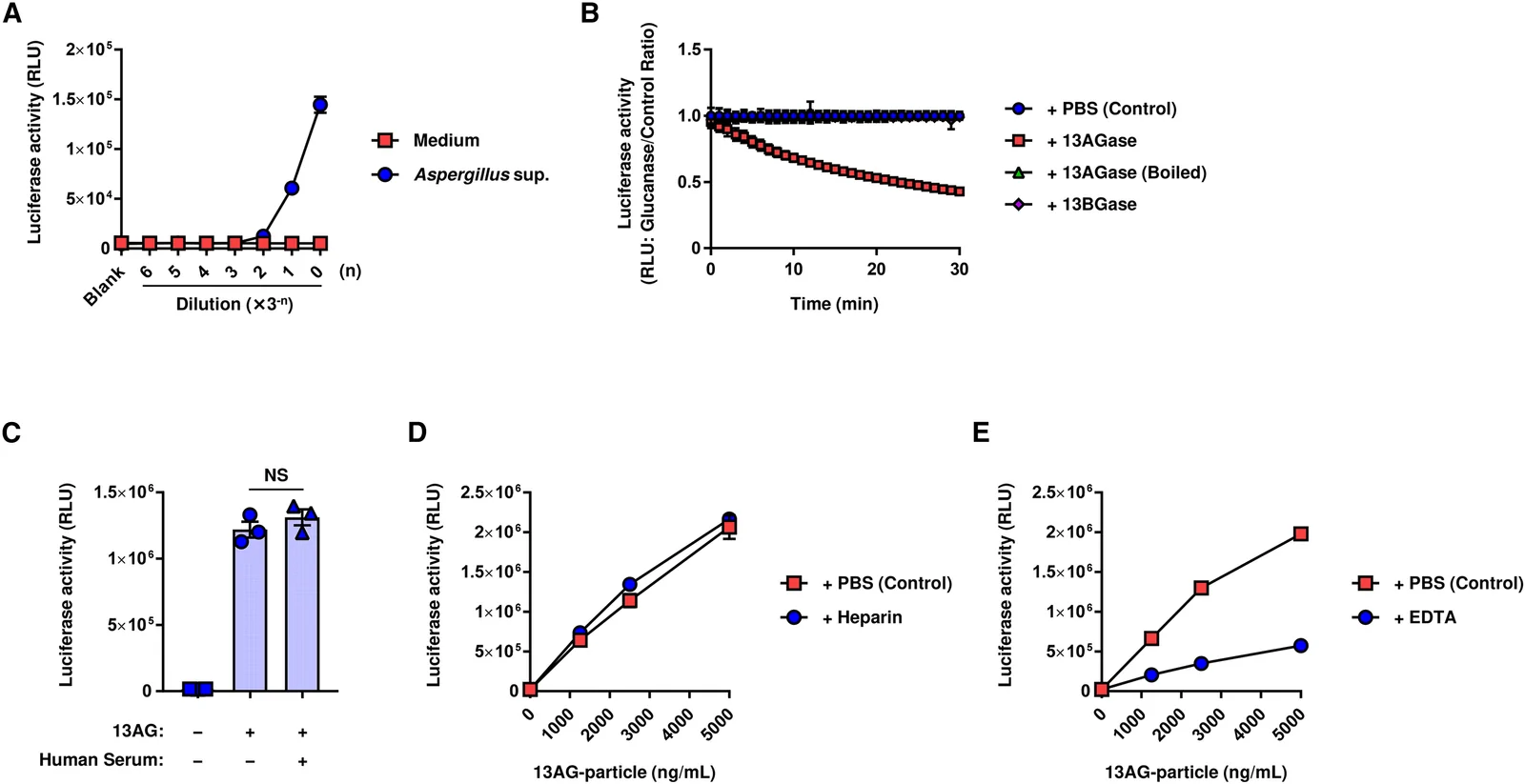
SLCA detection of soluble α-1,3-glucan structures in *Aspergillus* culture supernatants and compatibility with human serum (A) Reactivity of AglKA-CatD-D1090A-based SLCA toward 3-fold serial dilutions of *A. nidulans* culture supernatant. Uninoculated RPMI 1640 medium served as the medium control. (B) Enzymatic characterization of SLCA-reactive structures by real-time monitoring. *A. nidulans* culture supernatants were incubated with AglKA-CatD-D1090A-based SLCA probes for 30 min. PBS, active or heat-inactivated α-1,3-glucanase (13AGase), or β-1,3-glucanase (13BGase; specificity control) (0.1 μg/mL, final) was then added with the NanoLuc substrate and monitored for 30 min. Luminescence values were expressed relative to the PBS control (PBS control = 1). (C) Detection of α-1,3-glucan spiked into human serum or PBS after dilution and heat pretreatment. Data are presented as mean ± SEM of three technical replicates (*n* = 3). NS, not significant: no significant difference between PBS and serum (Student’s *t* test). (D and E) Effects of heparin (D) and EDTA (E) on the SLCA response. α-1,3-Glucan particles (0–5 μg/mL) were analyzed in PBS containing heparin (100 μg/mL) or EDTA (10 mM). (A, B, D, and E) Data are presented as mean ± SD of three technical replicates (*n* = 3). (A–E) Representative data from at least two independent experiments are shown. SLCA, split luciferase complementation assay.

To examine whether SLCA is compatible with human serum samples, α-1,3-glucan was spiked into serum and analyzed after dilution and heat pretreatment. The luminescent response from serum was comparable to that obtained from PBS, indicating that the serum matrix did not markedly interfere with AglKA-CatD-D1090A-based SLCA (Figure 5C). Additionally, we examined the effects of blood collection anticoagulants on the SLCA response. α-1,3-Glucan detection was not affected by heparin; however, the signal markedly reduced with EDTA, indicating that heparin is more compatible with SLCA-based detection (Figures 5D and 5E). These findings suggest that *Aspergillus* culture supernatants contain soluble, SLCA-reactive α-1,3-glucan structures and support the applicability of SLCA for their detection in human serum.

### Development of a highly sensitive ELISA for α-1,3-glucan detection

Finally, to overcome the limited sensitivity of SLCA at low ligand concentrations, we developed a luminescence-based sandwich ELISA using AglKA-CatD-D1090A-HiBiT as the detection probe (Figure 6A). For the capture probe, a Strep-tag II (ST2)-fused AglKA-CatD-D1090A construct was generated (Figure 6B), and its production and purity were confirmed using SDS-PAGE (Figure S1D). When the same *A. nidulans* culture supernatant used for SLCA was analyzed in 3-fold serial dilutions, the sandwich ELISA detected signals up to a 243-fold dilution (Figure 6C), whereas SLCA detected signals only up to a 9-fold dilution (Figure 5A). The control medium signals remained at background levels, indicating the substantially higher sensitivity of the sandwich ELISA.

**Figure 6:**
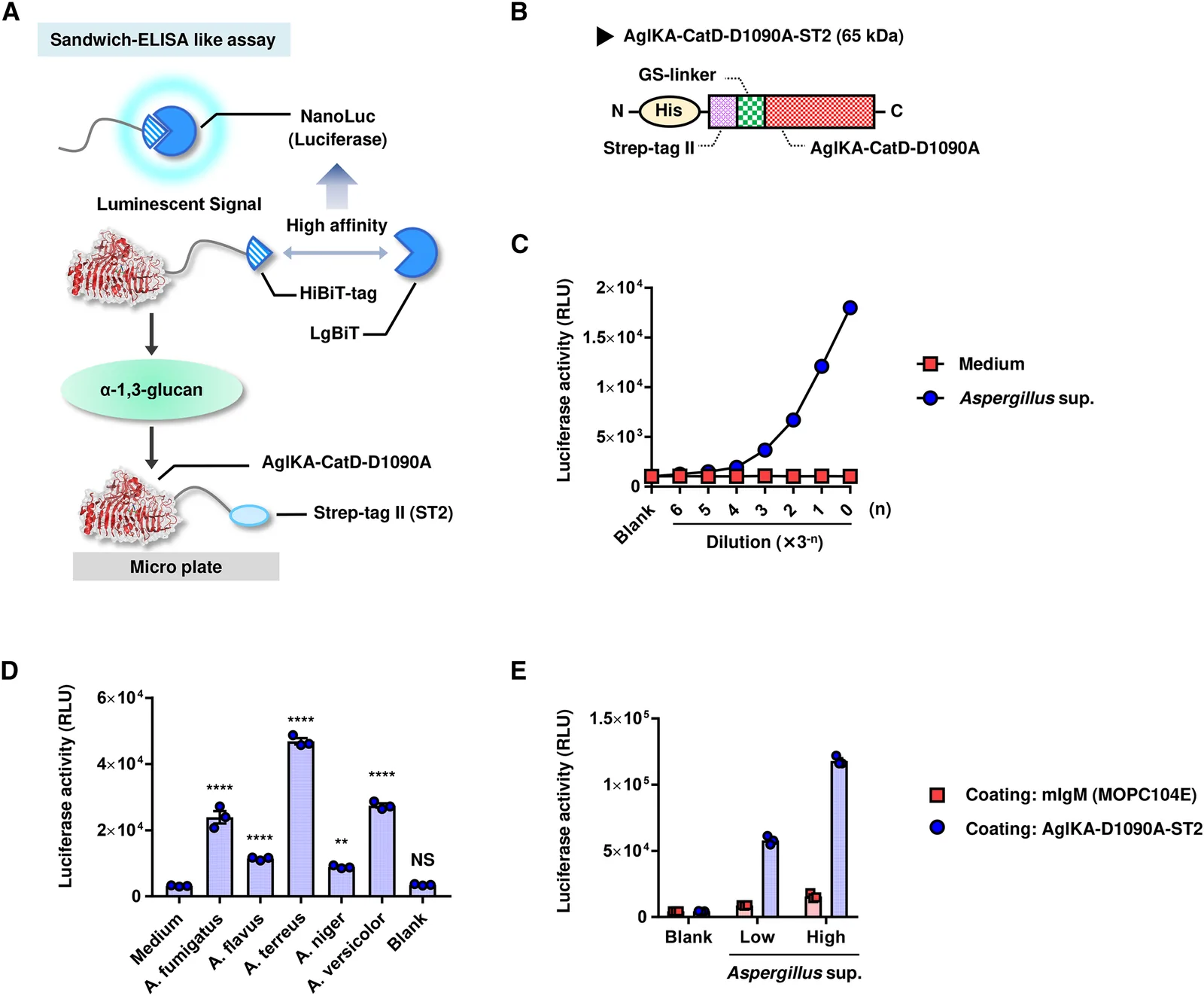
Highly sensitive detection of soluble α-1,3-glucan structures using an AglKA-CatD-D1090A-based sandwich ELISA (A) Schematic representation of the luminescence-based sandwich ELISA. (B) Design of the AglKA-CatD-D1090A-Strep-tag II construct used as the capture probe. (C) Detection of 3-fold serial dilutions of filtered *A. nidulans* culture supernatant using the AglKA-CatD-D1090A-based sandwich ELISA. RPMI 1640 medium without fungal inoculation was used as the medium control. Data are presented as mean ± SD of three technical replicates (*n* = 3). (D) Reactivity of the AglKA-CatD-D1090A-based sandwich ELISA toward 4-fold-diluted culture supernatants from *A. fumigatus*, *A. flavus*, *A. terreus*, *Aspergillus niger*, and *Aspergillus versicolor*. All culture supernatants and the RPMI 1640 medium control were filtered before analysis. Data are presented as mean ± SEM of three technical replicates (*n* = 3). ∗∗*p* < 0.01 and ∗∗∗∗*p* < 0.0001; NS, not significant: significant difference vs. control medium (Bonferroni’s multiple-comparisons test). (E) Comparison between AglKA-CatD-D1090A-ST2 and anti-α-1,3-glucan IgM MOPC104E as capture probes for detecting α-1,3-glucan structures. Undiluted (high) and three-fold-diluted (low) *A. nidulans* culture supernatant prepared in RPMI 1640 medium were analyzed using AglKA-CatD-D1090A-HiBiT as the detection probe. Data are presented as mean ± SEM of three technical replicates (*n* = 3). (C–E) Representative data from at least two independent experiments are shown.

Next, we examined culture supernatants from other *Aspergillus* species, including clinically important pathogens. All of the tested *Aspergillus* culture supernatants produced significantly higher luminescence signals than those of the control medium (Figure 6D).

To compare the performance of the AglKA-CatD-D1090A-based assay with that of a conventional α-1,3-glucan-binding probe, we conducted ELISA using the mouse monoclonal anti-α-1,3-glucan IgM antibody MOPC104E.^21^ This antibody-based assay showed only weak reactivity toward *Aspergillus* culture supernatants (Figure 6E). These results demonstrate that the AglKA-CatD-D1090A-based sandwich ELISA enables highly sensitive detection of soluble α-1,3-glucan structures released by clinically important *Aspergillus* species. Therefore, soluble α-1,3-glucan may serve as a complementary diagnostic biomarker for aspergillosis.

## Discussion

In this study, we developed AglKA-CatD-D1090A, a highly sensitive probe for α-1,3-glucan detection. Using this probe, soluble α-1,3-glucan structures were identified in culture supernatants from multiple *Aspergillus* species, and luminescence-based detection methods, including an SLCA and sandwich ELISA, were established. Therefore, these findings provide a basis for exploring *Aspergillus*-derived soluble α-1,3-glucan structures as complementary biomarkers for aspergillosis.

To date, only a limited number of α-1,3-glucan-binding probes, including a monoclonal antibody (i.e., clone MOPC104E) and an α-1,3-glucanase (AglKA)-derived CBM, have been developed.^22^^,^^36^ Under the conditions tested, the detection of soluble α-1,3-glucan structures in *Aspergillus* culture supernatants is weak with MOPC104E. Additionally, AglKA-CBM weakly binds to α-1,3-glucan on HKAN at the probe concentration used, despite its strong binding to lichen-derived isolichenan, an α-1,3-/α-1,4-glucan.^22^^,^^37^ Therefore, the sensitivity of conventional probes may not be sufficient for detecting soluble α-1,3-glucan structures under standard assay conditions. This limitation may have contributed to the limited attention given to extracellular soluble α-1,3-glucan structures.

The contrasting results obtained with AGN1 (GH71) and AglKA (GH87) highlight the importance of enzyme family and substrate-recognition mode in probe design. Wild-type AglKA and AGN1 generated different oligosaccharide products from α-1,3-glucan (Figure S2), suggesting that GH71 and GH87 enzymes engage the polysaccharide substrate in distinct ways. Consistently, AglKA-CatD variants lacking α-1,3-glucanase activity retained a strong binding activity, whereas binding with AGN1 mutants was not detected. Therefore, detectable substrate binding for α-1,3-glucanases is not simply a consequence of catalytic inactivation but also reflects the intrinsic substrate-engagement properties of the enzyme scaffold. AglKA-CatD-D1090A showed maximal binding at pH 8.0 and retained substantial binding at 50°C, corresponding to the reported optimal pH and temperature for parental AglKA catalytic activity.^22^

AglKA-CatD-D1090A could be successfully incorporated into the split-NanoLuc format, enabling construction of a homogeneous SLCA system. This assay allowed rapid, wash-free detection of α-1,3-glucan under various conditions. Given the roles of α-1,3-glucan in host adhesion and antifungal-associated cell-wall remodeling and the properties of conventional probes, these assays may facilitate studies of α-1,3-glucan dynamics during infection, disease progression, and therapy.^19^^,^^21^^,^^38^ The SLCA system enabled quantification of α-1,3-glucan exposure on conidia, where this polysaccharide is normally masked by the rodlet and melanin layers.^33^ Additionally, α-1,3-glucan structures were not limited to the fungal cell wall but are also released into the extracellular soluble fraction, similar to β-glucan and GM. Although their molecular form remains unclear, the requirement for at least three glucose units for ligand recognition and insolubility of α-1,3-glucan suggest that these structures may exist as soluble α-1,3-glucan-containing complexes. Their release could result from cell-wall remodeling or turnover. Association with other soluble cell-wall components may contribute to their apparent solubility, although their molecular composition and structural organization remain unknown. Previous nuclear magnetic resonance analyses of water-soluble fraction of *Aspergillus* also showed signals consistent with α-1,3-glucan. However, these signals were not discussed as evidence for extracellular soluble α-1,3-glucan structures.^39^

To explore the potential of α-1,3-glucan as a diagnostic biomarker for fungal infection, we established an AglKA-CatD-D1090A-based sandwich ELISA that enabled highly sensitive detection of soluble α-1,3-glucan structures. Using this assay, we found that this antigen was released by multiple clinically important *Aspergillus* species. α-1,3-Glucan synthase is also present in other pathogenic fungi, such as *Paracoccidioides*, *Histoplasma*, *Blastomyces*, and *Cryptococcus*; therefore, similar extracellular α-1,3-glucan structures may also be produced by these fungi.^21^^,^^40^^,^^41^^,^^42^ Additionally, AglKA-CatD-D1090A-based detection was compatible with human serum and heparin, supporting its potential use in diagnostic assay development. These findings support further evaluation of soluble α-1,3-glucan as a complementary diagnostic biomarker.

Although the presence, concentration, and diagnostic utility of α-1,3-glucan in clinical specimens remain to be established, our findings support its potential as a biomarker for aspergillosis and possible relevance to other α-1,3-glucan-producing fungal infections. Further studies are needed to determine the species specificity and clinical relevance. Collectively, AglKA-CatD-D1090A was established as a sensitive detection platform and provides a basis for developing α-1,3-glucan-based diagnostic approaches that may complement existing fungal diagnostics.

### Limitations of the study

Our findings establish a proof of concept; however, several limitations should be acknowledged. First, although we successfully demonstrated the detection of soluble α-1,3-glucan structures released into the culture supernatant of major clinically relevant *Aspergillus* species, their diagnostic performance has not yet been evaluated in clinical specimens. The concentration of soluble α-1,3-glucan in clinical specimens is currently unknown and may vary according to fungal burden, infecting species, disease stage, and antifungal treatment. Second, further improvement in sensitivity will be necessary to ensure reliable detection of low levels of soluble α-1,3-glucan structures. Finally, high-molecular-weight α-1,3-glucan are generally insoluble; therefore, the polysaccharide released by *Aspergillus* (the substance recognized by our probe) is presumed to exist in complexes with water-soluble molecules. However, its detailed molecular structure remains unclear. Future studies should focus on characterizing the molecular form of these released structures, further improving assay sensitivity, and evaluating their diagnostic performance in clinical specimens.

## Resource availability

### Lead contact

Requests for further information and resources should be directed to and will be fulfilled by the lead contact, Daisuke Yamanaka (ymnkd@toyaku.ac.jp).

### Materials availability

All unique/stable reagents generated in this study are available from the lead contact with a completed materials transfer agreement.

### Data and code availability

• All data reported in this paper will be shared by the lead contact upon request.
• This paper does not report original code.
• Any additional information required to reanalyze the data reported in this paper is available from the lead contact upon request.

## Acknowledgments

This work was supported by the 10.13039/501100001691Japan Society for the Promotion of Science (10.13039/501100001691JSPS) through a Grant-in-Aid for Scientific Research (B) (JSPS KAKENHI grant number JP23H02720) (to D.Y.), a Grant-in-Aid for 10.13039/501100001691JSPS Fellows (JSPS KAKENHI grant number JP26KJ1998) (to K.S.), and by the 10.13039/501100002241Japan Science and Technology Agency through the 10.13039/501100025019SPRING Program (JST 10.13039/501100025019SPRING, Japan grant number JPMJSP2134) (to K.S.). This work was also partially supported by 10.13039/100009619AMED and Tsukuba Clinical Research & Development Organization (T-CReDO) (grant number A23-12 to D.Y.) and by 10.13039/100008732The Uehara Memorial Foundation (to D.Y.). The authors also thank Editage (https://www.editage.com) for English language editing.

## Author contributions

Conceptualization, D.Y.; methodology, K.S. and D.Y.; investigation, K.S., M.K., K.M., and D.Y.; formal analysis, K.S. and D.Y.; writing – original draft, K.S. and D.Y.; writing – review and editing, M.K., K.M., Y.A., and D.Y.; funding acquisition, K.S. and D.Y.; resources, K.S., M.K., K.M., Y.A., and D.Y.; supervision, Y.A. and D.Y.

## Declaration of interests

K.S. and D.Y. are inventors on a pending patent related to this work.

## Declaration of generative AI and AI-assisted technologies in the manuscript preparation process

During the preparation of this work, the authors used ChatGPT (OpenAI) for language editing support. After using this tool or service, the authors reviewed and edited the content as needed and take full responsibility for the content of the publication.

## STAR★Methods

### Key resources table

**Table:** 

REAGENT or RESOURCE	SOURCE	IDENTIFIER
**Antibodies**		

Anti-His-tag mAb-Alexa Fluor 488	MBL	Cat# D291-A48; RRID: AB_10695477
Purified Mouse IgM λ	BioLegend	Cat# 401101

**Bacterial and virus strains**		

*Escherichia coli* DH5α	BioDynamics Laboratory Inc.	Cat# DS230
*Escherichia coli* SHuffle	New England Biolabs	Cat# C3029J
*Streptococcus mutans* NBRC 13955	NBRC	NBRC 13955

**Biological samples**		

Human Serum, Pooled	MP Biomedicals	Cat# 2930149

**Chemicals, peptides, and recombinant proteins**		

Bovine Serum Albumin	Sigma-Aldrich	Cat# A7030-100G
Fluorescent Brightener 28 (CFW)	Sigma-Aldrich	Cat# F3543
Laminarin	Sigma-Aldrich	Cat# L9634
Mannan	Sigma-Aldrich	Cat# M3640
Isolichenan	ELICITYL	Cat# GLU601
Dextran T10	Pharmacia	N/A
Biotin Dextran	Invitrogen	Cat# D1956
Tween 20	FUJIFILM Wako	Cat# 166–21213
Starch	FUJIFILM Wako	Cat# 195–03961
Curdlan	FUJIFILM Wako	Cat# 032–09902
Chitin	FUJIFILM Wako	N/A
D-glucose	FUJIFILM Wako	Cat# 049–31165
Sucrose	FUJIFILM Wako	Cat# 193–09545
Heparin sodium	FUJIFILM Wako	N/A
Citric Acid	FUJIFILM Wako	Cat# 035–03495
Disodium Hydrogenphosphate Dodecahydrate	FUJIFILM Wako	Cat# 196–02835
0.5M-EDTA Solution (pH 8.0)	Nacalai Tesque	Cat# 06894–14
Pustulan	InvivoGen	Cat# tlrl-pst
Nigerose	Megazyme	Cat# O-NGR
Nigerotriose	Megazyme	Cat# O-NGR3
Nigerotetraose	Megazyme	Cat# O-NGR4
Nigeropentaose	Megazyme	Cat# O-NGR5
Nigerohexaose	Megazyme	Cat# O-NGR6
Xylan	Megazyme	Cat# P-ACXYL
Chitosan oligosaccharides	Tokyo Chemical Industry	Cat# C2762
Tween 80	Tokyo Chemical Industry	Cat# T0546
Zymolyase 100T	Seikagaku Corporation	Cat# 120493
Potato Dextrose Broth	Becton, Dickinson and Company	Cat# 254920
Potato Dextrose Agar	Becton, Dickinson and Company	Cat# 213400
Brain Heart Infusion Broth	Shimadzu Diagnostics Corporation	Cat# 05509
RPMI 1640 medium	Gibco	Cat# C11875500BT
GtfB-His	This paper	N/A
AglKA-CBM-HiBiT	This paper	N/A
AglKA-CBM-SmBiT	This paper	N/A
AglKA-CatD-WT-His	This paper	N/A
AglKA-CatD-WT-HiBiT	This paper	N/A
AglKA-CatD-D1067A-HiBiT	This paper	N/A
AglKA-CatD-D1090A-HiBiT	This paper	N/A
AglKA-CatD-D1090A-SmBiT	This paper	N/A
AglKA-CatD-D1090A-LgBiT	This paper	N/A
AglKA-CatD-D1090A-Strep tag II	This paper	N/A
AglKA-CatD-D1091A-HiBiT	This paper	N/A
AGN1-WT-HiBiT	This paper	N/A
AGN1-D259N-HiBiT	This paper	N/A
AGN1-E262Q-HiBiT	This paper	N/A

**Critical commercial assays**		

PrimeSTAR Max Premix (2×)	Takara Bio	Cat# SD1960
5× In-Fusion HD Enzyme Premix	Takara Bio	Cat# ST0345
TALON Metal Affinity Resin	Takara Bio	Cat# Z5501N
c-Pagel HR	ATTO	Cat# CHR520L
FastGene Gel/PCR Extraction Kit	Nippon Genetics	Cat# FG-91202
Maxwell 16 Cell LEV DNA Purification Kit	Promega	Cat# AS1140
Nano-Glo HiBiT Lytic Detection System	Promega	Cat# N3030
Nano-Glo Luciferase Assay System	Promega	Cat# N1110
QuickBlue Staining Solution	BioDynamics Laboratory Inc.	Cat# DS500
QuickGene Plasmid Kit S II	Kurabo Industries Ltd.	Cat# PL-S2
Quick Start Bovine Serum Albumin Standard Kit	Bio-Rad	Cat# 5000207
Quick Start Bradford Reagent	Bio-Rad	Cat# 5000205

**Experimental models: Organisms/strains**		

*Aspergillus terreus* NBRC 6123	NBRC	NBRC 6123
*Aspergillus nidulans* NBRC 8003	NBRC	NBRC 8003
*Aspergillus flavus* NBRC 33021	NBRC	NBRC 33021
*Aspergillus fumigatus* NBRC 33022	NBRC	NBRC 33022
*Aspergillus niger* NBRC 33023	NBRC	NBRC 33023
*Aspergillus versicolor* NBRC 33027	NBRC	NBRC 33027
*Candida albicans* NBRC 1385	NBRC	NBRC 1385
*Rhizopus oryzae* NBRC 9364	NBRC	NBRC 9364
*Aspergillus fumigatus* AfS35	Miyazawa et al.^35^	N/A
*Aspergillus fumigatus* AfS35-Δ*ags1*Δ*ags*2Δ*ags3*	Miyazawa et al.^35^	N/A

**Oligonucleotides**		

Primers for amplification of enzyme genes, see Table S1	This paper	N/A

**Recombinant DNA**		

Plasmid: GtfB	This paper	N/A
Plasmids: AglKA-CBM	This paper	N/A
Plasmids: AglKA-CatD	This paper	N/A
Plasmids: AGN1	This paper	N/A

**Software and algorithms**		

GraphPad software	GraphPad Software	https://www.graphpad.com/

**Other**		

Maxwell 16	Promega	AS1000
GloMax Discover System	Promega	GM3000
EVOS Imaging System	Thermo Fisher Scientific	EVOS M5000
BLItz system	Pall ForteBio Inc.	N/A

### Experimental model and study participant details

#### Construction of expression plasmids

DNA fragments encoding AglKA- and AGN1-derived constructs were codon-optimized for *E. coli* and synthesized. For GtfB, *Streptococcus mutans* Clarke 1924 NBRC 13955 [NITE biological resource center (NBRC, Chiba, Japan)] was statically cultured overnight at 37°C in brain-heart infusion broth. Genomic DNA was purified using the Maxwell 16 Cell LEV DNA Purification Kit (Promega, Madison, WI, USA), and the signal peptide-deleted region of *gtfB*, encoding glucosyltransferase-I (EC 2.4.1.5), was amplified by PCR using PrimeSTAR Max DNA Polymerase (Takara Bio, Shiga, Japan) according to the manufacturer’s instructions. PCR and gel-extracted products were purified using the FastGene Gel/PCR Extraction Kit (Nippon Genetics, Tokyo, Japan).

A modified pCold vector backbone was generated from pCold I DNA (Takara Bio, Shiga, Japan; GenBank: AB186388.1) by joining nucleotide regions 1–300 and 361–4407 and inserting a DNA fragment encoding a GS linker (GGSGGGSGGSG) between these regions. Where indicated, sequences encoding HiBiT, SmBiT, or Strep-tag II (ST2) were inserted upstream of the GS linker to generate N-terminal tag-fusion constructs; constructs without these tags contained only the N-terminal His tag followed by the GS linker. DNA fragments encoding GtfB (Asp39–Asn1476), AglKA-CBM (Ala31–Ala466), AglKA-CatD (Asn720–Gln1293)-, or AGN1 (Asp21–Phe424)-derived proteins were inserted into the modified pCold vector using the In-Fusion cloning system (Takara Bio). For the LgBiT-fusion construct, the LgBiT sequence was joined to the C-terminal end of the AglKA-CatD-D1090A coding sequence. Point mutations were introduced using site-directed mutagenesis with the appropriate primer pairs.

Plasmids were transformed into *E. coli* DH5α (BioDynamics Laboratory Inc., Tokyo, Japan), which was cultured in LB medium containing ampicillin. Plasmids were purified from *E. coli* using the QuickGene Plasmid Kit S II (KURABO Industries Ltd., Osaka, Japan). The sequences of all final plasmids were confirmed by DNA sequencing performed by Eurofins Genomics K.K. (Tokyo, Japan).

#### Protein expression and purification

Plasmids were transformed into *E. coli* SHuffle (New England Biolabs Inc., MA, USA), which was pre-cultured in LB medium supplemented with ampicillin at 37°C. Protein expression was then induced at 15°C, followed by the addition of isopropyl-β-d-thiogalactopyranoside (0.1 mM) and cultivation for an additional 16–48 h. Cells were washed in PBS containing phenylmethylsulphonyl fluoride (0.2 mM) and dithiothreitol (1 mM) and sonicated (50-watt, 30 s) three times on ice. The supernatant was applied to a TALON resin (Takara Bio) column and washed with PBS containing 15 mM imidazole. His-tagged recombinant proteins were eluted with 150 mM imidazole and dialyzed against PBS using a 3.5-kDa cutoff membrane. Protein concentrations were determined using a Bradford assay kit (Quick Start Dye and bovine serum albumin [BSA] standard; Bio-Rad, CA, USA). Purified recombinant proteins were separated using SDS-PAGE with 5–20% c-PAGEL HR precast gradient gels (ATTO, Tokyo, Japan) and stained with QuickBlue Staining Solution (BioDynamics Laboratory Inc., Tokyo, Japan).

#### Preparation of fungal samples

*Aspergillus terreus* NBRC 6123, *A. nidulans* NBRC 8003, *A. flavus* NBRC 33021, *A. fumigatus* NBRC 33022, *A. niger* NBRC 33023, *A. versicolor* NBRC 33027, *C*. *albicans* NBRC 1385, and *Rhizopus oryzae* NBRC 9364 were obtained from NBRC and maintained on potato dextrose agar (PDA). *A. fumigatus* AfS35 and AfS35-Δ*ags1*Δ*ags2*Δ*ags3* were previously generated and described.^35^ For mycelial sample preparation, these strains were cultured in RPMI 1640–MOPS medium at 37°C with shaking at 200 rpm for 24 h, washed with sterile water, autoclaved (121°C, 20 min), and freeze-dried.

Spores were collected from PDA slants using PBS containing 0.05% Tween 80 and used either untreated or after heating at 90°C for 10 min. *C. albicans* colonies (37°C, 24 h) were suspended in PBS at 1 × 10^8^ cells/mL and heated at 90°C for 10 min and used as test samples.

For preparation of mycelial samples and culture supernatants, spore suspensions were inoculated into 5 mL of liquid medium at 1 × 10^6^ spores/mL. For HKAN preparation, *A. nidulans* spores were cultured in PD medium at 37°C with shaking at 200 rpm for 3 days. Additionally, spores of the NBRC filamentous fungal strains were cultured in RPMI 1640 medium at 37°C with shaking at 200 rpm for 7 days. Mycelia and culture supernatants were separated using centrifugation (6000 × g, 10 min). Mycelia were washed with water, autoclaved (121°C, 20 min), washed again, freeze-dried, suspended in PBS at 1 mg/mL, and dispersed with bead beating. Culture supernatants were heated at 90°C for 10 min and filtered through a 0.45-μm membrane.

### Method details

#### Direct ELISA

The isolichenan (1 μg/mL, PBS, 25 μL)-coated or mock-coated (PBS) 96-well white plate (Greiner Bio-One, Kremsmunster, Austria) was blocked with 50 μL of PBS-containing 1% BSA and 0.05% Tween 20 (assay buffer) and then incubated with the HiBiT-tagged proteins (1 μg/mL, assay buffer, 25 μL) for 30 min. After washing with PBS-containing 0.05% Tween 20 (wash buffer), a mixture of free LgBiT and NanoLuc substrate (Promega) were added (25 μL), and HiBiT tag-derived luminescence was measured using a GloMax Discover microplate reader (Promega).

#### Pull-down-based binding assay

HiBiT-tagged proteins (1 μg/mL, 5 μL) in assay buffer were mixed with 25 μL of a heat-killed *A. nidulans* (HKAN) mycelial suspension (10 mg/mL, assay buffer), or PBS (control) in a microtube and incubated at room temperature (23°C) for 10 min. To assess the ligand-recognition specificity of the probe, HKAN suspension (20 mg/mL, 200 μL) in PBS was incubated with active or heat-inactivated wild-type α-1,3-glucanase (AglKA-CatD-WT-His; 1 μg/mL, final) overnight at 37°C. The heat-inactivated glucanase was prepared using heating at 90°C for 10 min before use. After incubation, the reaction mixtures were heated at 90°C for 10 min to terminate enzymatic activity. After washing three times with 150 μL of assay buffer, the suspension was mixed with free LgBiT and NanoLuc substrate (30 μL) in assay buffer and transferred to a 96-well white plate (Greiner Bio-One), and luminescence was measured using a GloMax Discover microplate reader (Promega).

#### Fluorescence microscopy

HKAN mycelial suspensions (1 mg/mL, 50 μL) in PBS were pretreated with active or heat-inactivated α-1,3-glucanase (AglKA-CatD-WT-His; 5 μg/mL, final) at 37°C for 30 min. The reaction mixtures were heated at 90°C for 10 min to stop the enzymatic reaction. The pretreated HKAN samples were then incubated with AglKA-CatD-D1090A-HiBiT (10 μg/mL) at room temperature (23°C) for 30 min. After washing with PBS, Alexa Fluor 488 (AF488)-conjugated anti-His-tag mAb and Fluorescent Brightener 28 (calcofluor white; 2.5 μg/mL each) were added and incubated at room temperature (23°C) for 30 min. After washing, the samples were transferred to a 384-well clear plate (Greiner Bio-One), and images were captured using an EVOS Imaging System (Thermo Fisher Scientific, MA, USA).

#### Enzymatic synthesis of α-1,3-glucan

α-1,3-Glucan was enzymatically synthesized using recombinant GtfB based on previously reported methods.^29^^,^^43^ Sucrose (20% w/v) was incubated with recombinant GtfB (10 μg/mL, final) in PBS at 30°C for 48 h. Depending on the intended application, α-1,3-glucan preparations were synthesized either without dextran to obtain α-1,3-glucan particles or with dextran (0.17% w/v) as an acceptor/primer for GtfB-catalyzed α-glucan synthesis to improve sample handling in immobilization-based assays. To prepare biotinylated α-glucan for BLI analysis, commercially available biotinylated dextran was added to the reaction mixture in place of dextran, allowing the synthesized α-glucan material to be immobilized on streptavidin sensor tips through the biotinylated dextran moiety. The resulting α-1,3-glucan particles or soluble fractions were collected, treated with ethanol, and dried with acetone. Prior to use, the products were resuspended in PBS and dispersed using bead beating (for ELISA and SLCA) or sonication (for BLI analysis).

#### Fluorophore-assisted carbohydrate electrophoresis

Synthesized α-1,3-glucan particle (1 mg/mL) was incubated with HiBiT-tagged AglKA-CatD or AGN1 proteins, including wild-type and mutant forms (10 μg/mL, final), at 37°C for 24 h. The reactions were terminated by heating at 90°C for 5 min, and the reducing ends of the degradation products were labeled with 8-aminonaphthalene-1,3,6-trisulfonic acid (Invitrogen, CA, USA) and separated using 40% PAGE, as described by Wakita et al. (2017).^44^ Glucose and nigero-oligosaccharides (DP2–6) were run as molecular size markers.

#### BLI

Biotinylated α-glucan in PBS (4 μL) was immobilized on a streptavidin sensor chip for 120 s. Binding data were collected using BLI using a BLItz system (Pall ForteBio Inc., CA, USA). BLI measurements were performed in BLI buffer consisting of PBS containing 0.1% BSA and 0.005% Tween 20, which was also used for baseline equilibration (30 s) and HiBiT-tagged probe dilution. AglKA-CBM and AglKA-CatD variants were compared at 100 nM, whereas AglKA-CatD-D1090A was analyzed using 2-fold serial dilutions (12.5–200 nM) to determine the association rate constant (*k*_a_), dissociation rate constant (*k*_d_), and equilibrium dissociation constant (*K*_D_) as previously described by Yamanaka et al. (2020).^24^

#### Competitive ELISA

Synthesized α-glucan (5 μg/mL, PBS) was immobilized on a 96-well white plate (Greiner Bio-One) and processed as described for the direct ELISA. AglKA-CatD-D1090A-HiBiT (100 ng/mL) was pre-incubated for 30 min with glucose, nigero-oligosaccharides (250 μM), or the same synthesized α-glucan as that for competitors (500 μg/mL). The mixtures were added to the plate and incubated for 30 min, followed by HiBiT luminescence measurement.

#### Stability assessment of AglKA-CatD-D1090A

The stability of AglKA-CatD-D1090A was evaluated using direct ELISA with synthesized α-glucan-coated plates (5 μg/mL). For thermal stability, AglKA-CatD-D1090A-HiBiT was diluted in PBS, heated at 20°C–90°C for 5 min, cooled to room temperature, and mixed with an equal volume of assay buffer before application to the plate (0.5 μg/mL, final). For pH stability, AglKA-CatD-D1090A-HiBiT was diluted in McIlvaine buffer^45^ containing 1% BSA at the indicated pH values and applied to the plate (0.5 μg/mL, final). HiBiT-derived luminescence was measured as described for the direct ELISA.

#### SLCA

A mixture containing SmBiT- and LgBiT-fused glycan-binding probes in assay buffer (200 nM each, 5 μL) was mixed with the sample solution (10 μL) in a 96-well white plate (Greiner Bio-One) and incubated with shaking at room temperature (23°C) for 30 min. The NanoLuc substrate (15 μL) in assay buffer was then added, and luminescence from reconstituted NanoLuc was measured using a GloMax luminometer (Promega).

#### Human serum spike-in assay

Synthesized α-1,3-glucan was spiked into human serum or PBS at 5 μg/mL. Each sample was then diluted 5-fold with PBS, heated at 90°C for 10 min, and used as the test sample for the SLCA.

#### Sandwich ELISA

AglKA-CatD-D1090A-ST2 or anti-α-1,3-glucan IgM MOPC104E (2 μg/mL in PBS) was coated onto a 96-well white plate (Greiner Bio-One) as the capture probe. After blocking and washing, sample solutions were added and incubated for 1 h. The plate was then washed and incubated with AglKA-CatD-D1090A-HiBiT (1 μg/mL) for 1 h as the detection probe. After washing, HiBiT-derived luminescence was measured using free LgBiT and substrate as described above.

### Quantification and statistical analysis

All statistical analyses were performed using GraphPad Prism 7.0 (GraphPad Software, CA, USA). Data are presented as mean ± SD or SEM, as indicated in the corresponding figure legends. Based on the results of the normality test, significant differences were analyzed using a two-tailed unpaired Student’s *t* test or Welch’s *t* test and one-way ANOVA followed by multiple comparisons using the Bonferroni’s test. The exact value of n and what n represents, including whether n represents technical replicates or independent biological replicates, are specified in the corresponding figure legends. Details of the statistical tests used for individual experiments are also provided in the corresponding figure legends. Statistical significance was defined as *p* < 0.05. Significance is indicated as follows: ∗*p* < 0.05, ∗∗*p* < 0.01, ∗∗∗*p* < 0.001, ∗∗∗∗*p* < 0.0001.
