# Supplementary material for: An engineered α-1,3-glucanase-derived probe enables sensitive detection of α-1,3-glucan and reveals its soluble release by Aspergillus
Source: iScience. 2026 Oct 26;29(11):117952. doi: 10.1016/j.isci.2026.117952 (PMC13734777; doi:10.1016/j.isci.2026.117952)
Supplement: Supplementary file 1 [file mmc1.pdf]

iScience, Volume *29*

Supplemental information

An engineered α-1,3-glucanase-derived probe enables sensitive detection of α-1,3-glucan and reveals its soluble release by *Aspergillus*

Kento Suzuki, Masahiro Kimura, Ken Miyazawa, Yoshiyuki Adachi, and Daisuke Yamanaka

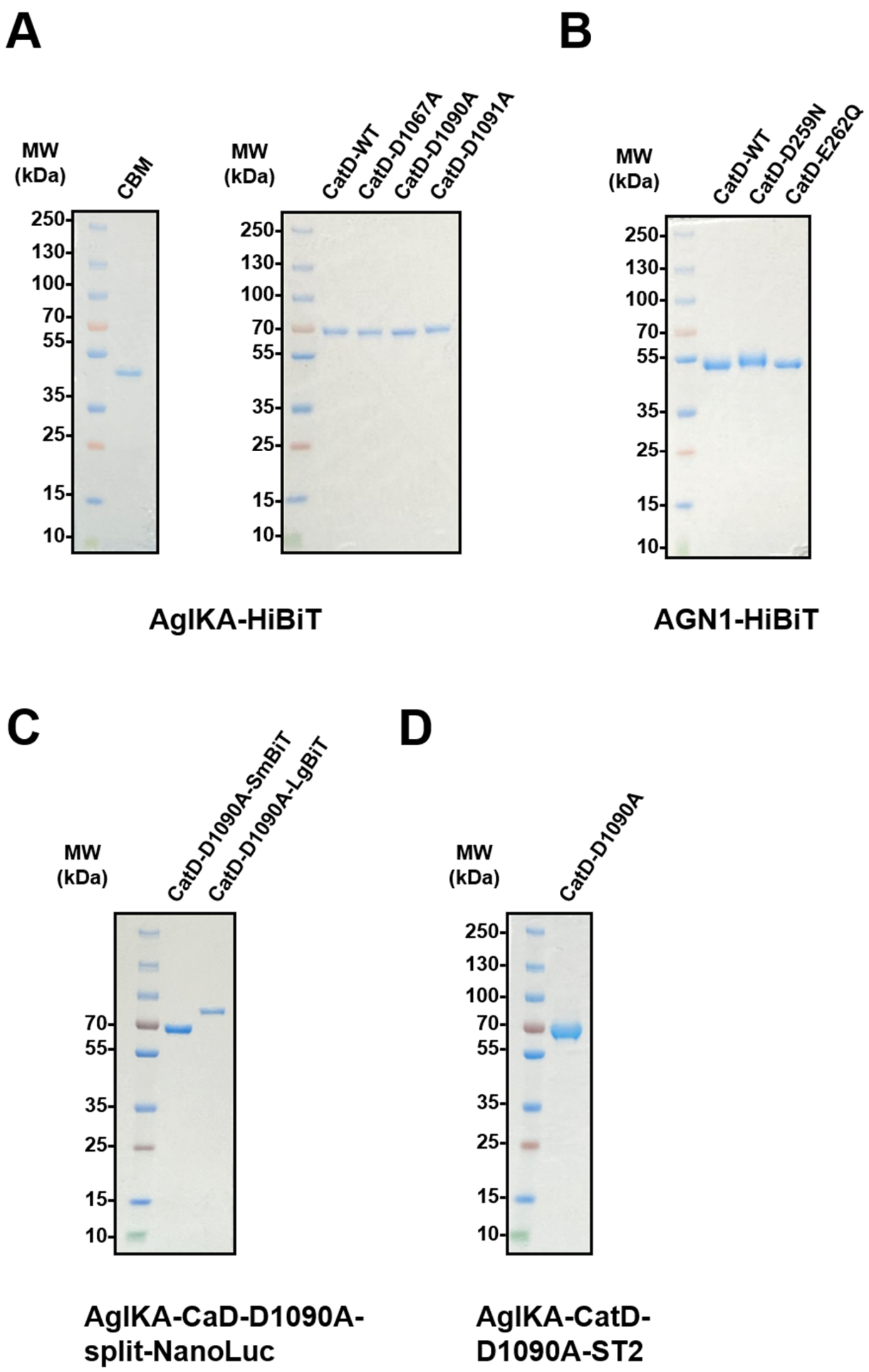

Figure S1. SDS-PAGE of recombinant proteins used in this study

(A) AglKA-HiBiT, related to Figure 1. (B) AGN1-HiBiT, related to Figure 1. (C) AglKA-CatD-D1090A-Split-NanoLuc, related to Figure 3. (D) AglKA-CatD-D1090A-ST2, related to Figure 6. WT, wild type.

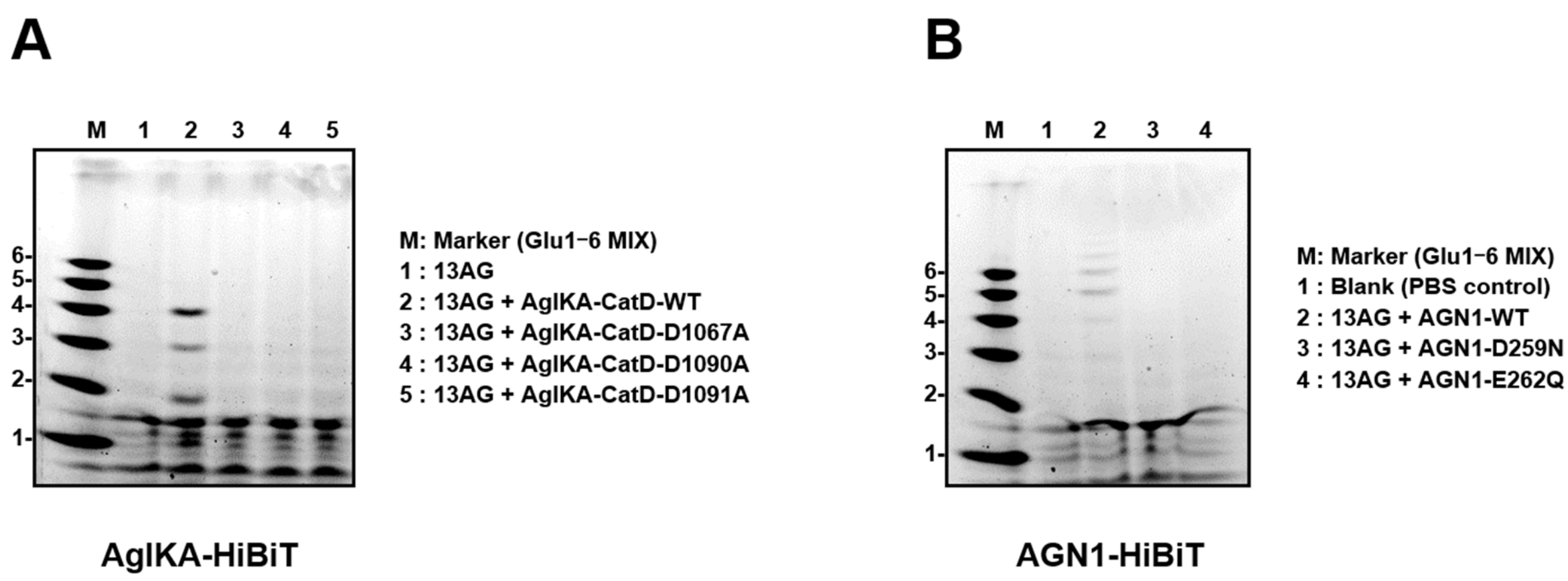

Figure S2. FACE analysis of α-1,3-glucan degradation by AglKA and AGN1, related to Figure 1

α-1,3-Glucan particles were incubated with (A) AglKA-CatD-HiBiT or (B) AGN1-HiBiT proteins at 37°C for 24 h, and the resulting degradation products were fluorescently labeled with ANTS and analyzed using FACE. WT, wild type; Marker, glucose/nigero-oligosaccharides (DP2–6) mixture from 1 mM stocks; ANTS, 8-aminonaphthalene-1,3,6-trisulfonic acid; FACE, fluorophore-assisted carbohydrate electrophoresis

Table S1. Primers used in this study

Primer name	Sequence (5'→3')
GTFB-Fw	GGTGGATCAGGTGGTGATTCTAATGAATCGAAATC
GTFB-Rv	AGCAGAGATTACCTAGTTAATCCGAACTCGTTCTC
AgIKA-WT-Fw	AACGGCGATGATGGCCTGGCGGTGTG
AgIKA-WT-Rv	GCCATCATCGCCGTTATTCCGGATGC
AgIKA-D1067A-Fw	CTGGCCGCCGGTGTTAATTTCGCTCA
AgIKA-D1067A-Rv	AACACCGGCGGCCAGATTGTTACGAA
AgIKA-D1091A-Fw	GGCGATGCTGGCCTGGCGGTGTGGAC
AgIKA-D1091A-Rv	TCCGGAATAACGGCGATGCTGGCCTG
AGN1-WT-Fw	TTATGGCGAGTCCCATTATATCGGAA
AGN1-WT-Rv	TGGGACTCGCCATAATCGTTCCAGGT
AGN1-D259N-Fw	CTGGAACAATTATGGCGAGTCCCATT
AGN1-D259N-Rv	CCATAATTGTTCCAGGTTAGAACCTC
AgIKA-CBM-Fw	GGTGGATCAGGTGGTCGGGAGGTCCGAATC
AgIKA-CBM-Rv	AGCAGAGATTACCTACGCAGTAACCGGAATTG
AgIKA-CatD-N-Ins-Fw	GGTGGATCAGGTGGTAATCTAGTCGTATATGC
AgIKA-CatD-N-Ins-Rv	AGCAGAGATTACCTACTGAATGGTCAGGTTG
AgIKA-CatD-C-Ins-Fw	CATATCGAAGGTAGGAGTCGTATATGCACA
AgIKA-CatD-C-Ins-Rv	ACCGCCACTTCCACCTGGTCAGGTTGAAGC
AGN1-N-Ins-Fw	GGTGGATCAGGTGGTGATAAGATGGTTGTTG
AGN1-N-Ins-Rv	AGCAGAGATTACCTAAAAGTAAAGCACACCAG
